# Integrated Evolutionary and Multi-Omic Analysis of STAT Family Activation Across Solid Tumors

**DOI:** 10.3390/genes17050547

**Published:** 2026-05-03

**Authors:** Dunja Lukic, Pietro Hiram Guzzi, Federico Manuel Giorgi

**Affiliations:** 1 Department of Pharmacy and Biotechnology, University of Bologna, Via Gobetti 85, 40129 Bologna, Italy; 2 Department of Surgical and Medical Sciences, Magna Graecia University of Catanzaro, 88100 Catanzaro, Italy; hguzzi@unicz.it; 3 Data Analytics Research Center, University of Catanzaro, 88100 Catanzaro, Italy

**Keywords:** STAT, pan-cancer, transcriptomics, master regulator analysis

## Abstract

**Background/Objectives**: The STAT (Signal Transducer and Activator of Transcription) family of seven transcription factors mediates cytokine and growth-factor signaling, regulating proliferation, differentiation, and immunity. While STAT3/STAT5 are established oncogenes and STAT1/STAT2 are classically viewed as tumor suppressors, emerging evidence indicates context-dependent roles in tumorigenesis. This study aimed to integrate evolutionary analysis with bulk transcriptomic, regulon, single-cell, and exploratory chromatin-binding analyses of the STAT family in human solid tumors. **Methods**: Orthologs and paralogs of human STAT genes (81 sequences total) were retrieved across vertebrates and invertebrates; a phylogenetic tree was constructed using MUSCLE alignment and Neighbor-Joining in MEGA12. Differential expression was assessed in TCGA solid tumors versus GTEx normal tissues. Master-regulator activity was inferred using the *corto* algorithm. Single-cell RNA-seq datasets were used to compare malignant and non-malignant cell populations. STAT1 chromatin binding was examined via ChIP-seq in interferon-stimulated HeLa and K562 cells. **Results**: Phylogeny resolved seven conserved vertebrate clades, with endocrine-responsive STAT3/STAT5 showing higher conservation and immune-associated STAT1/STAT2/STAT4/STAT6 exhibiting faster divergence. The majority of STAT genes were frequently upregulated across multiple solid tumors, with activated regulons confirming functional transcriptional engagement. Single-cell analysis demonstrated tumor-cell-autonomous upregulation of STAT1 and STAT2 in the HNSCC dataset. STAT1 ChIP-seq revealed asymmetric forward/reverse-strand read density around peak summits, supporting non-canonical DNA recognition. **Conclusions**: The STAT family operates as an evolutionarily conserved, broadly activated transcriptional module in human solid cancers, combining quantitative upregulation with qualitative shifts in DNA-binding dynamics. These findings refine our understanding of JAK/STAT signaling in oncology and highlight opportunities for network-targeted therapies.

## 1. Introduction

The Signal Transducer and Activator of Transcription (STAT) family comprises seven distinct genes in the human genome: STAT1, STAT2, STAT3, STAT4, STAT5A, STAT5B, and STAT6 [1]. These transcription factors are integral to the transmission of signals from extracellular cytokines and growth factors to the nucleus. They play an essential role in regulating critical physiological processes, including cellular proliferation, differentiation, apoptosis, and immune system modulation [2]. The canonical activation of STAT proteins is primarily mediated by Janus kinases (JAKs) [3,4]. Upon ligand binding to cell-surface receptors, JAKs auto-phosphorylate and subsequently phosphorylate specific tyrosine residues on the recruited STAT monomers [5,6]. This phosphorylation event triggers STAT dimerization via their highly conserved Src homology 2 (SH2) domains, followed by rapid nuclear translocation and DNA binding to specific regulatory elements [7].

In the context of oncology, the dysregulation of STAT signaling contributes directly to tumorigenesis across multiple cancer types [8]. The molecular mechanisms driving this dysregulation, however, show a stark dichotomy between hematological malignancies and solid tumors. In blood cancers, particularly T-cell large granular lymphocytic (T-LGL) leukemia and natural killer/T-cell lymphomas, somatic gain-of-function mutations in the SH2 domains of STAT3 and STAT5B act as primary oncogenic drivers, occurring in up to 30% to 50% of patients [9,10]. These mutations directly induce constitutive phosphorylation, dimerization, and unconstrained proliferation. Conversely, somatic mutations in STAT genes are exceptionally rare in solid tumors [2]. Instead, solid tumors primarily exploit the constitutive activation and quantitative transcriptomic upregulation of wild-type STAT proteins [11]. STAT3 and STAT5 (comprising STAT5A and STAT5B) are classically defined as oncogenes [12,13]. Elevated expression and hyperactivation of STAT3 have been documented in a diverse array of solid tumors, including gastric cancer, glioblastoma, prostate cancer, and hepatic cancer, where they consistently correlate with poor patient prognosis and decreased overall survival [14]. In breast cancer, STAT3 activity is frequently associated with highly aggressive and dedifferentiated tumor subtypes, while STAT5 activity is more often linked to lower-grade tumors [15,16].

Conversely, STAT1 and STAT2 have traditionally been characterized as tumor suppressors, primarily due to their roles as the main mediators of interferon (IFN) signaling, which typically elicits anti-proliferative and pro-apoptotic responses. However, recent data suggest a context-dependent role. For instance, in advanced prostate cancer, elevated STAT1 expression has been implicated in mediating cellular resistance to both docetaxel chemotherapy and radiation therapy [17]. STAT1 can also act as an oncogene and tumor promoter in specific contexts, such as leukemia and breast cancer, where its upregulation drives cancer cell proliferation, metastasis, and immune evasion [18,19]. This duality highlights the need to evaluate the entire STAT family network simultaneously, rather than studying individual members in isolation.

The oncogenic potential of STAT proteins is largely driven by their capacity to transcriptionally regulate networks of genes essential for cell cycle progression, survival, and immunosuppression. STAT3 directly activates the transcription of Cyclin D1, c-Myc, and anti-apoptotic members of the Bcl-2 family (such as Bcl-xL and Mcl-1), while concomitantly repressing pro-apoptotic factors [20]. In glioblastoma, the aberrant activation of the IL-6/JAK/STAT3 axis not only drives rapid cellular proliferation but also heavily alters the tumor microenvironment, rendering it immunosuppressive and facilitating immune evasion [21]. Furthermore, STAT3 can interact with other oncogenic transcription factors, such as the Nuclear factor erythroid 2-related factor 2 (NRF2), to synergistically promote tumor progression and chemoresistance [22]. In addition to classical transcription activation, unphosphorylated STAT3 (u-STAT3) has also been shown to drive the expression of critical oncogenic and inflammatory mediators, such as IL-6, IL-8, CCL5, and the MET oncogene, further diversifying its pro-tumorigenic repertoire [23].

The complexity of STAT signaling in tumorigenesis has been extensively studied utilizing animal models, spanning from *Drosophila melanogaster* to transgenic mice, highlighting the deep evolutionary roots and conserved nature of these pathways [24]. Indeed, understanding the conserved oncogenic functions of STAT genes also requires an evolutionary perspective. The STAT gene family emerged early in metazoan evolution, with functional orthologs identified in invertebrates such as the nematode *Caenorhabditis elegans* [25]. The *Caenorhabditis elegans* STAT ortholog STA-1 lacks the N-terminal oligomerization domain present in vertebrate STATs, suggesting that this structural feature was acquired later in evolution to provide additional regulatory control over spatial arrangement and oligomerization [25]. During early vertebrate evolution, the STAT repertoire expanded rapidly from a single ancestral gene to six members, likely driven by two rounds of whole-genome duplication. The subsequent tandem duplication of STAT5 in early Eutherian mammals gave rise to the modern seven-member family found in humans. Phylogenetic analyses reveal a distinct evolutionary dichotomy: STAT proteins operating downstream of endocrine hormones (STAT3, STAT5A, STAT5B) exhibit higher sequence conservation, whereas those mediating immune signals (STAT1, STAT2, STAT4, STAT6) display a significantly faster evolutionary divergence rate [26].

The functional output of STAT proteins is ultimately governed by their DNA-binding specificities. Activated STAT dimers recognize and bind to conserved DNA consensus sequences, such as Gamma-activated sequence (GAS) elements and Interferon-stimulated response elements (ISRE) [7,27]. The advent of chromatin immunoprecipitation coupled with high-throughput sequencing (ChIP-seq) has provided high-resolution, genome-wide maps of STAT binding [28,29]. For example, ChIP-seq profiling of STAT1 in interferon-stimulated HeLa cells identified tens of thousands of putative binding regions, revealing a massive scale of genomic interaction [30]. Surprisingly, transcriptome profiling coupled with these ChIP-seq datasets demonstrated that only a fraction of the genes harboring STAT1 binding sites are actually transcriptionally modulated, hinting at the complexity of STAT-mediated gene regulatory networks [31]. Furthermore, structural and biochemical studies indicate that STAT proteins can bind non-canonical or half-site elements under some conditions [27]. These observations suggest additional complexity in STAT DNA recognition beyond canonical GAS binding [32].

Despite extensive work on individual STAT members [1,4,33], an integrated family-level analysis spanning evolution, bulk transcriptomics, regulon activity, and single-cell expression in solid tumors remains limited. Here, we combine phylogenetic reconstruction, bulk differential expression, master-regulator analysis, and single-cell RNA-seq to characterize STAT family behavior across human solid tumors. We also include an exploratory STAT1 ChIP-seq analysis to examine whether peak-centered read distributions show reproducible asymmetry.

## 2. Materials and Methods

### 2.1. Sequence Retrieval and Phylogenetic Analysis

We retrieved orthologs and paralogs of human STAT genes as follows. All human gene symbols in the manuscript and in the pipeline are defined by HGNC (HUGO Gene Nomenclature Committee) [34], while non-human ortholog names follow the nomenclature adopted by the NCBI (National Center for Biotechnology Information) database. First, we included in the list of human STATs the following genes: STAT1, STAT2, STAT3, STAT4, STAT5A, STAT5B, STAT6. Then, we defined orthologs using the OrthoDB database queried from the HGNC HCOP Orthology Prediction web interface [35]. We searched for orthologs, co-orthologs, and paralogs in the following species: Human, Chimp, Macaque, Mouse, Rat, Dog, Cat, Horse, Cattle, Pig, Opossum, Platypus, Chicken, Anole lizard, *Xenopus tropicalis*, Zebrafish, Fruit fly, and *Caenorhabditis elegans*. We found 74 genes that were in an orthologous or co-orthologous relationship with the human STAT genes, so together with the 7 human STATs, our analysis defined 81 genes. For each gene, we retrieved a protein isoform as follows: when available, a curated RefSeq was selected (with “NP” identifier), and when multiple sequences were available, the longest was chosen. All sequence accession numbers are reported in the analysis. The retrieval was performed using R scripts and the *rentrez* package within the R statistical environment [36].

Protein sequences were aligned using the MUSCLE algorithm [37] with default parameters, including a gap open penalty of −2.90, a gap extend penalty of 0.00, and a hydrophobicity multiplier of 1.20. Sequence clustering during the alignment phase was performed using the UPGMA method. Phylogenetic reconstruction was subsequently carried out in MEGA12 utilizing the Neighbor-Joining (NJ) method [38]. Evolutionary distances were computed using the Poisson substitution model, assuming uniform rates among sites and applying pairwise deletion for gaps and missing data. Topological support was evaluated using a bootstrap test with 1000 replicates.

### 2.2. RNA-Seq Analysis

To investigate the transcriptomic landscape of STAT genes across solid tumors, we utilized harmonized bulk RNA sequencing data from The Cancer Genome Atlas (TCGA) [39] and the Genotype-Tissue Expression (GTEx) project [40]. To account for study-specific biases and batch effects between these two repositories, the dataset was preprocessed using the Wang normalization pipeline [41]: this harmonization reduces cross-cohort technical bias but does not eliminate differences related to tissue composition, tumor purity, or residual batch effects. We restricted our analysis to 15 tumor types that possessed matched normal tissue data from GTEx: BLCA (Bladder Urothelial Carcinoma), BRCA (Breast Invasive Carcinoma), COAD (Colon Adenocarcinoma), ESCA (Esophageal Carcinoma), KIRC (Kidney Renal Clear Cell Carcinoma), KIRP (Kidney Renal Papillary Cell Carcinoma), LIHC (Liver Hepatocellular Carcinoma), LUAD (Lung Adenocarcinoma), LUSC (Lung Squamous Cell Carcinoma), PRAD (Prostate Adenocarcinoma), READ (Rectum Adenocarcinoma), STAD (Stomach Adenocarcinoma), THCA (Thyroid Carcinoma), UCEC (Uterine Corpus Endometrial Carcinoma), and UCS (Uterine Carcinosarcoma). Statistical analyses were conducted within the R environment (version 4.4.2). Differential gene expression contrasting tumor versus normal tissues was evaluated using the DESeq2 package (version 1.48.1) [42]. To assess the functional activation of STAT-driven transcriptional networks, we applied the *corto* algorithm (version 1.2.4) [43]. Regulatory networks were inferred using 200 bootstraps. Edge significance was set to a *p*-value of 10^−10^ for most datasets, with the exception of the UCS cohort; due to its substantially lower sample size (n = 47), a threshold of *p* = 0.01 was utilized to ensure the resulting networks were comparable in scale to the other cohorts as in [44]. All *p*-values were corrected for multiple testing using the Benjamini–Hochberg procedure. Only STAT genes with a network of at least 10 inferred targets are shown in the figures.

### 2.3. Single-Cell RNA-Seq Analysis

To examine whether bulk-expression patterns were also detectable at single-cell resolution, we interrogated the head and neck squamous cell carcinoma (HNSCC) dataset GSE103322 [45]. This specific dataset was selected due to its high-resolution profiling of the tumor microenvironment (TME) and robust pre-existing annotations, which reliably distinguish malignant epithelial cells from surrounding stromal and immune populations. The data, comprising 21,883 genes across 5902 individual cells, was queried and retrieved using the TMExplorer R package v1.18.0 [46], chosen specifically for its efficiency in accessing and formatting standardized single-cell TME datasets. Downstream processing was performed utilizing the Seurat package v5.4.0 [47]. Cells were grouped into “Cancer” and “Non-Cancer” compartments according to the original dataset annotation. The expression matrix underwent log-normalization, followed by the identification of 2000 highly variable features. Data scaling and Principal Component Analysis (PCA) were performed, and the first 15 principal components were utilized to generate Uniform Manifold Approximation and Projection (UMAP) embeddings for visualization. Statistical comparisons of STAT expression between the Cancer and Non-Cancer cell populations were conducted using a one-sided Wilcoxon rank-sum test, with *p*-values subsequently adjusted via the Benjamini–Hochberg method. To validate our findings across diverse tumor microenvironments, three additional single-cell RNA-seq datasets were acquired as Gene Expression Omnibus Series (GSE): GSE72056 (melanoma) [48], GSE75688 (breast cancer) [49], and GSE150430 (nasopharyngeal carcinoma) [50]. These datasets were processed, normalized, and analyzed utilizing the same Seurat-based pipeline described above to quantify STAT family expression in malignant versus non-malignant single cells.

### 2.4. ChIP-Seq Analysis

To assess the genomic binding dynamics of STAT1, we analyzed chromatin immunoprecipitation sequencing (ChIP-seq) data derived from interferon-stimulated HeLa S3 cells (GEO accession: GSE12782 [51]). To assess whether similar read-distribution patterns were also visible in a second dataset from a different cellular context, we additionally processed STAT1 ChIP-seq data from the K562 human chronic myelogenous leukemia cell line (GEO accession: GSE31477; sample GSM935472 [52]). Both ChIP-seq experiments utilized a total STAT1 antibody. Raw FASTQ files were aligned to the human reference genome using HISAT2 [53], and the resulting alignments were sorted and indexed utilizing SAMtools [54]. Peak calling was executed with MACS3 [55] using a q-value threshold of 0.05, with summit identification enabled, the building model bypassed, and an extension size of 200 bp. Genomic loci of interest, such as the IRF9, STAT2, and ICAM1 promoters, were visualized using the Integrative Genomics Viewer (IGV) [56], with the “color by read strand” option. To evaluate the strand-specific read distribution and investigate potential spatial binding asymmetries of the STAT1 dimer at target sites, we extracted sequence alignment information directly from the BAM files within the R environment. We calculated and plotted the density of forward- and reverse-mapped reads relative to the center of the identified MACS3 peak summits, generating an aggregated profile globally across the entire experiment.

## 3. Results

### 3.1. Evolutionary Expansion and Lineage-Specific Diversification of the STAT Family

**Figure 1:**
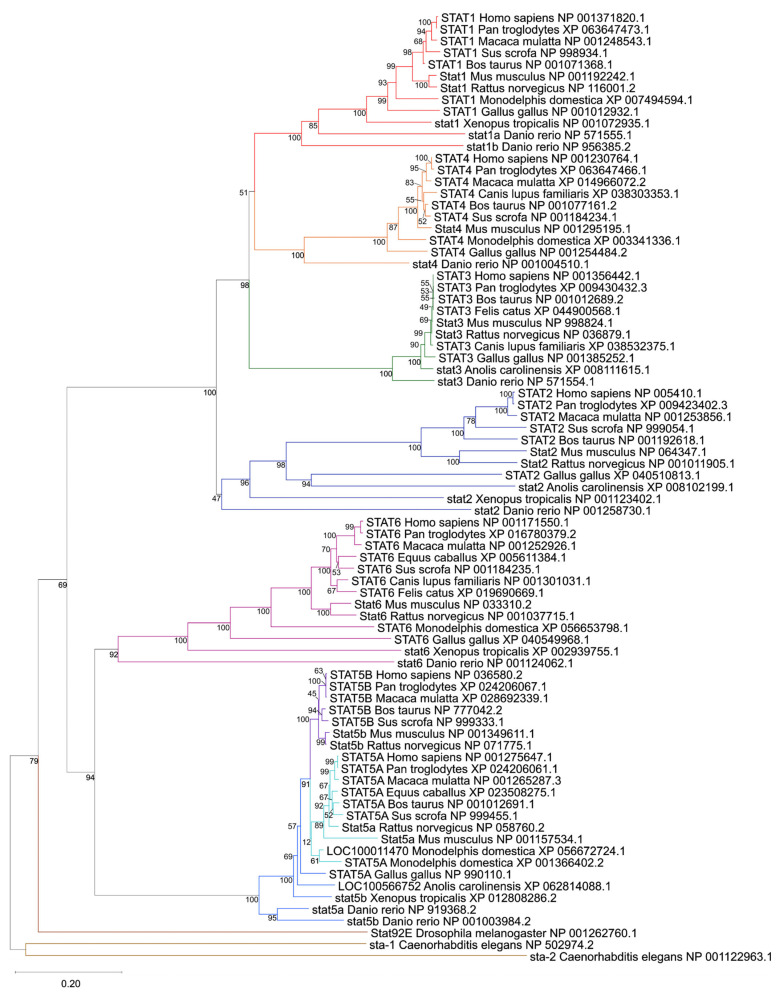
Phylogenetic tree of STAT proteins inferred from 81 protein sequences (derived via OrthoDB) spanning vertebrates and selected invertebrates. The tree was constructed using Neighbor-Joining with Poisson-corrected distances and 1000 bootstrap replicates. Distinct clades corresponding to STAT1 (red), STAT2 (dark blue), STAT3 (green), STAT4 (orange), STAT5A (cyan), STAT5B (purple), STAT6 (magenta). Invertebrate sequences from *Drosophila melanogaster* (Stat92E) and *Caenorhabditis elegans* (sta-1 and sta-2), highlighted in brown and tan color, respectively, belong to distinct outgroups, supporting a single ancestral STAT origin followed by vertebrate expansion. Bootstrap support values are indicated at internal nodes. The scale bar represents the number of amino acid substitutions per site. Lineage-specific duplications (e.g., teleost stat1a/stat1b and marsupial STAT5A-like sequences) are visible within respective clades.

The phylogenetic reconstruction of 81 STAT-related protein sequences derived via OrthoDB across vertebrate and invertebrate species resolved seven major clades corresponding to human STAT1, STAT2, STAT3, STAT4, STAT5A, STAT5B, and STAT6 (Figure 1). Invertebrate STAT orthologs from *Drosophila melanogaster* (Stat92E) and *Caenorhabditis elegans* (sta-1, sta-2) clearly form an external branch, supporting their role as ancestral outgroups and confirming the monophyletic expansion of vertebrate STATs from a single ancestral gene [24,25,57].

Within vertebrates, each human STAT gene clustered with its corresponding orthologs across mammals, birds, reptiles, amphibians, and fish, supported by robust bootstrap values at the principal nodes. STAT3, STAT5A, and STAT5B form deeply conserved branches with short interspecies branch lengths, consistent with strong purifying selection acting on endocrine and growth-factor–responsive STATs [26,58].

In contrast, immune-associated STATs (STAT1, STAT2, STAT4, STAT6) display longer branch lengths and greater divergence across vertebrate clades, suggesting accelerated evolutionary dynamics, likely reflecting adaptation to pathogen-driven immune pressures [24,26]. This dichotomy between endocrine and immune STAT subclasses is clearly visible in the tree topology (Figure 1).

The tree reveals several lineage-specific duplication and loss events. Teleost *Danio rerio* contains two STAT1 paralogs (stat1a and stat1b), clustering within the STAT1 clade but forming distinct sub-branches, consistent with teleost-specific whole-genome duplication events [59]. Similarly, the marsupial *Monodelphis domestica* exhibits two STAT5A-like sequences (canonical STAT5A and LOC100011470), indicative of a lineage-specific duplication. It is important to note that while we deliberately retained the official NCBI nomenclature for all analyzed sequences to ensure database consistency, our phylogenetic topology strongly supports the biological reclassification of several uncharacterized loci. For instance, the clustering clearly identifies LOC100566752 in *Anolis carolinensis* and LOC100011470 in *Monodelphis domestica* as STAT5 genes. An evaluation of the genomic synteny of these loci on Chromosome 2 (NC_077228.1) of *Monodelphis domestica* confirms that the canonical STAT5A and LOC100011470 sequences are positioned in direct tandem proximity to each other (Supplementary Figure S1). This finding reinforces that this is a lineage-specific localized duplication event distinct from the broader eutherian genomic expansion.

Together, these findings reinforce a model in which early vertebrate whole-genome duplications expanded the STAT repertoire from a single ancestral gene to six members, followed by tandem duplication of STAT5 in eutherian mammals and additional lineage-restricted events. The preserved orthology across mammals, combined with selective paralog expansions in fish and marsupials, underscores both functional conservation and adaptive diversification within the STAT family.

### 3.2. Pan-Cancer Upregulation of STAT Transcription Factors

**Figure 2:**
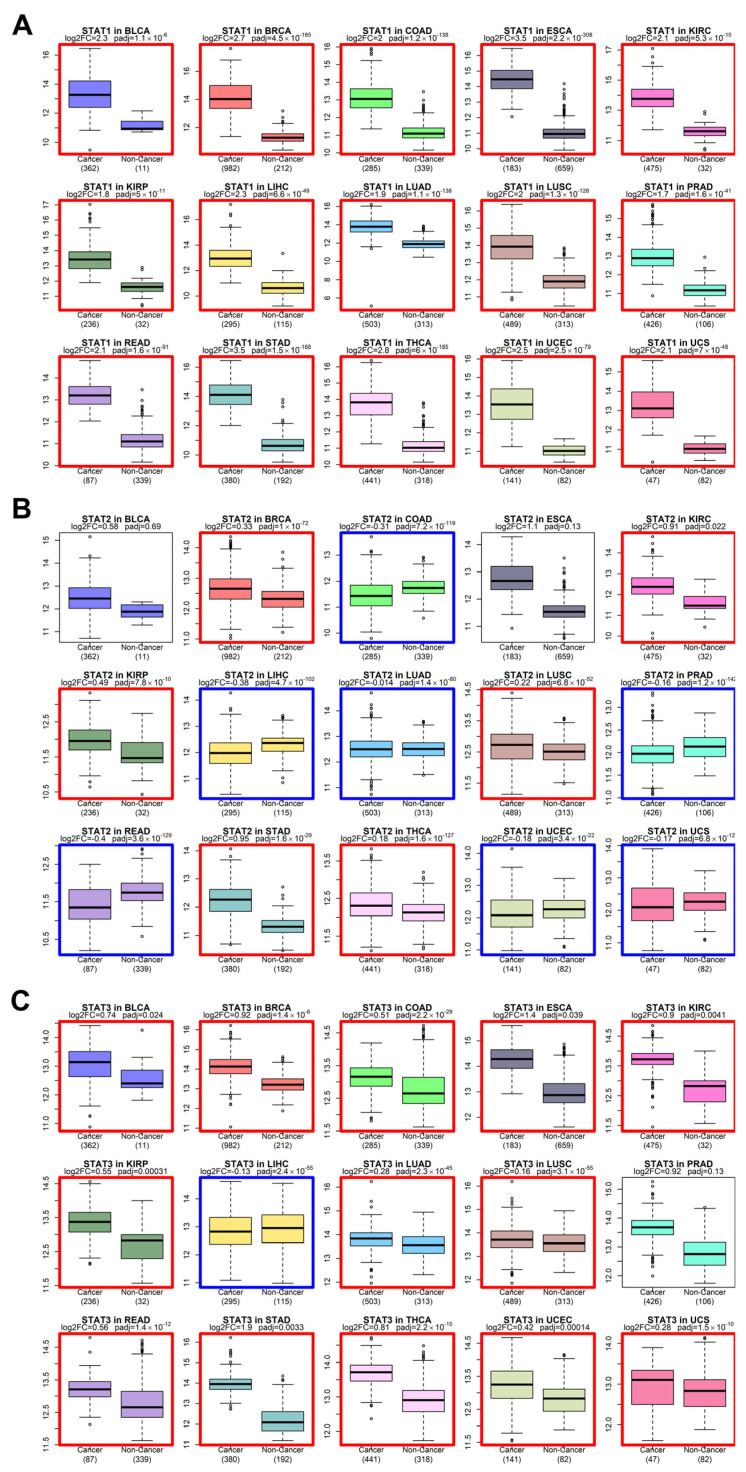
Differential expression analysis of STAT genes comparing tumor (TCGA) and corresponding normal tissue (GTEx) samples across multiple cancer types. Expression values represent normalized log-transformed RNA-seq counts. (**A**) STAT1 expression across tumor types. (**B**) STAT2 expression. (**C**) STAT3 expression. Each panel contains multiple boxplots corresponding to individual cancer types (BLCA, BRCA, COAD, ESCA, KIRC, KIRP, LIHC, LUAD, LUSC, PRAD, READ, STAD, THCA, UCEC, UCS). For each cancer type, the left box represents tumor samples (Cancer) and the right box represents non-cancer tissues (Non-Cancer). Sample sizes are indicated below each comparison. Center lines represent medians; boxes indicate interquartile ranges; whiskers denote distribution spread; outlier dots represent individual samples. Adjusted *p*-values are displayed above each comparison. Across most tumor types, STAT family members exhibit consistent upregulation in cancer relative to normal tissues. A colored box surrounds each comparison when a significant change (adjusted *p*-value < 0.05) occurs: red when the gene is upregulated in cancer, and blue when it is downregulated.

**Figure 3:**
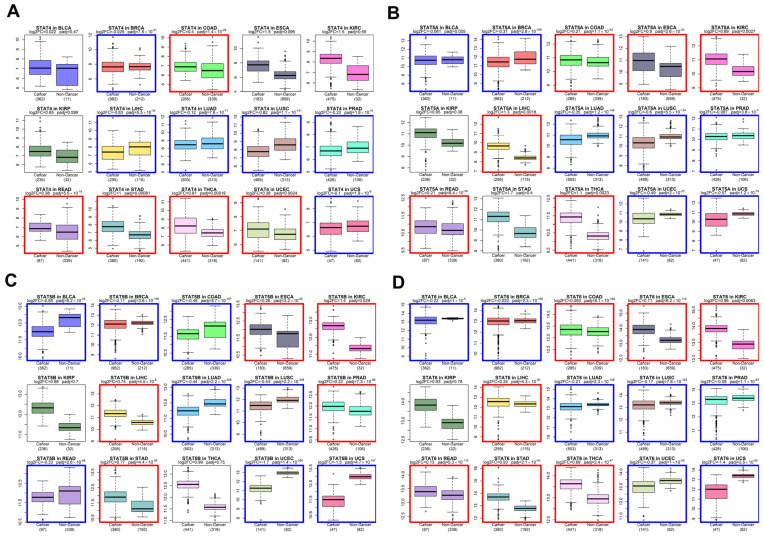
Differential expression analysis of STAT genes comparing tumor (TCGA) and corresponding normal tissue (GTEx) samples across multiple cancer types. Expression values represent normalized log-transformed RNA-seq counts. (**A**) STAT4 expression. (**B**) STAT5A expression. (**C**) STAT5B expression. (**D**) STAT6 expression. Each panel contains multiple boxplots corresponding to individual cancer types (BLCA, BRCA, COAD, ESCA, KIRC, KIRP, LIHC, LUAD, LUSC, PRAD, READ, STAD, THCA, UCEC, UCS). For each cancer type, the left box represents tumor samples (Cancer) and the right box represents non-cancer tissues (Non-Cancer). Sample sizes are indicated below each comparison. Center lines represent medians; boxes indicate interquartile ranges; whiskers denote distribution spread; outlier dots represent individual samples. Adjusted *p*-values are displayed above each comparison. Across most tumor types, STAT family members exhibit consistent upregulation in cancer relative to normal tissues. A colored box surrounds each comparison when a significant change (adjusted *p*-value < 0.05) occurs: red when the gene is upregulated in cancer, and blue when it is downregulated.

We next examined whether the evolutionary conservation of STAT genes is paralleled by recurrent expression changes in solid tumors. Using harmonized bulk RNA-seq datasets from TCGA tumors and GTEx normal tissues [39,40,41], we performed differential expression analysis across multiple solid cancer types (Figure 2 and Figure 3).

Across the majority of tumor cohorts analyzed—including BLCA, BRCA, COAD, ESCA, KIRC, LUAD, LUSC, STAD, and others—STAT family members exhibited consistent upregulation in tumors relative to corresponding normal tissues. While STAT3 and STAT5 genes have historically been considered canonical oncogenes [12], our pan-cancer analysis demonstrates that the upregulation phenomenon extends broadly to STAT1, STAT2, STAT4, and STAT6.

STAT1 and STAT2, traditionally associated with interferon-mediated tumor suppression [1], were frequently elevated in tumor tissues. This pattern supports a context-dependent functional shift, where chronic interferon signaling may contribute to tumor adaptation, immune modulation, or therapy resistance rather than pure tumor suppression [18]. STAT6 and STAT4, typically associated with Th2 and immune polarization pathways [60], also showed elevated expression in multiple solid tumors, suggesting a broader rewiring of cytokine-responsive transcriptional programs in cancer.

The directionality of differential expression was consistent across tumor types, pointing to a systemic activation of STAT-mediated transcription rather than isolated tumor-specific events.

### 3.3. STAT Transcriptional Networks Are Functionally Activated in Cancer

Gene expression upregulation does not necessarily equate to transcriptional activity. Therefore, we evaluated STAT functional activity using a master regulator analysis implemented with the *corto* algorithm [43]. *Corto* infers regulatory networks based on co-expression and data processing inequality principles, enabling estimation of transcription factor activity independently of its mRNA abundance.

**Figure 4:**
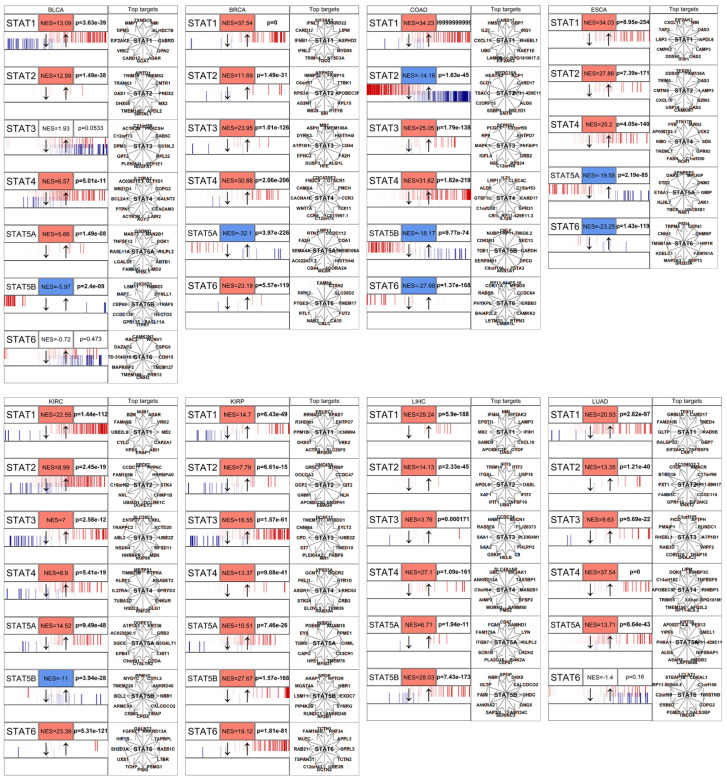
*Corto*-based master regulator analysis comparing STAT regulon activity between cancer and non-cancer samples across multiple tumor cohorts. For each tumor type (organized in grid layout), normalized enrichment scores (NES) and corresponding *p*-values are shown for individual STAT family members. The black arrows simply indicate the direction of the horizontal box (left genes are more downregulated, right genes are more upregulated). Red color gradients indicate positive enrichment (regulon activation in cancer), whereas blue gradients indicate negative enrichment (reduced activity in cancer). Small barcodes represent distribution of target gene differential expression across the ranked gene list. Network diagrams illustrate inferred STAT-centered regulatory networks, with edges connecting STAT factors to predicted target genes. Plots are organized alphabetically and show results for 8 tumors (BLCA, BRCA, COAD, ESCA, KIRC, KIRP, LIHC, LUAD). Across many tumor types, STAT regulons show coordinated activation, confirming functional transcriptional engagement beyond simple mRNA upregulation.

**Figure 5:**
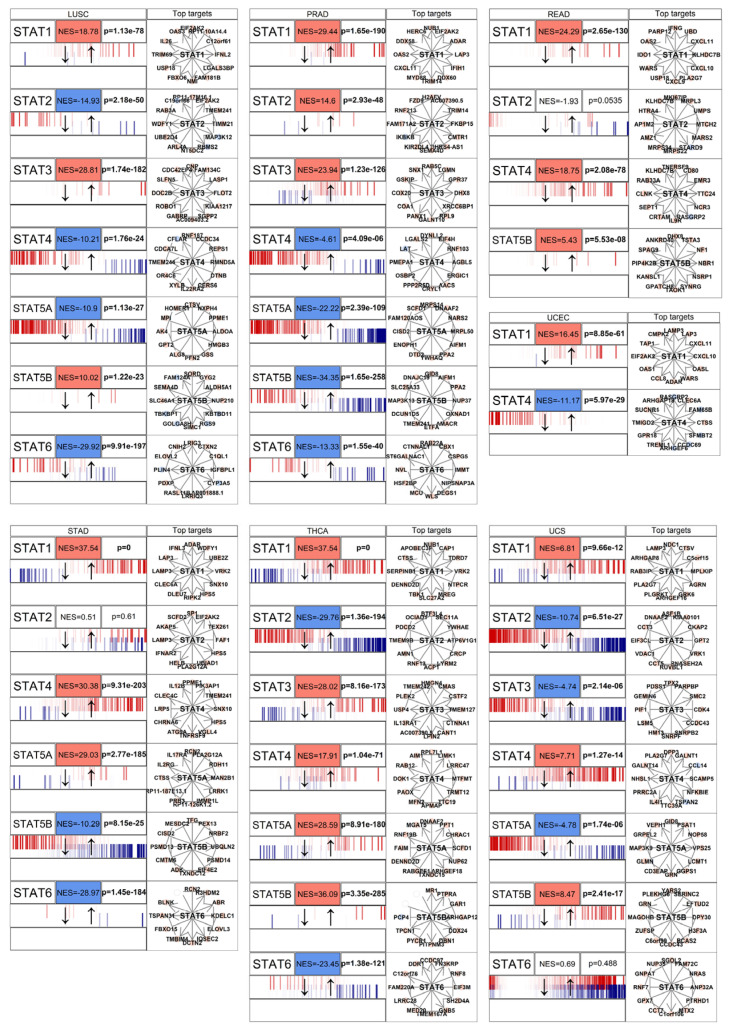
*Corto*-based master regulator analysis comparing STAT regulon activity between cancer and non-cancer samples across multiple tumor cohorts. For each tumor type (organized in grid layout), normalized enrichment scores (NES) and corresponding *p*-values are shown for individual STAT family members. The black arrows simply indicate the direction of the horizontal box (left genes are more downregulated, right genes are more upregulated). Red color gradients indicate positive enrichment (regulon activation in cancer), whereas blue gradients indicate negative enrichment (reduced activity in cancer). Small barcodes represent distribution of target gene differential expression across the ranked gene list. Network diagrams illustrate inferred STAT-centered regulatory networks, with edges connecting STAT factors to predicted target genes. Plots show results for 7 tumor types (LUSC, PRAD, READ, STAD, THCA, UCEC, UCS).

Across solid tumor types, STAT-driven regulons showed significant positive enrichment scores (NES), indicating coordinated upregulation of downstream targets (Figure 4 and Figure 5). This observation demonstrates that STATs are not merely transcriptionally elevated but are acting as active master regulators within oncogenic gene networks.

In multiple tumor contexts, STAT1, STAT3, and STAT6 exhibited particularly strong regulon activation, consistent with their known roles in regulating genes involved in cell cycle progression, inflammatory signaling, and immune evasion [33]. The concordance between mRNA upregulation and regulon activation supports a model of sustained pathway activation rather than passive overexpression.

Notably, STAT network activation was often broader than individual gene upregulation, suggesting cooperative or compensatory behavior among STAT paralogs. This reinforces the concept that STATs function as an interconnected transcriptional module rather than independent actors.

### 3.4. Single-Cell Validation of STAT1, STAT2, and STAT6 Upregulation in Malignant Cells

**Figure 6:**
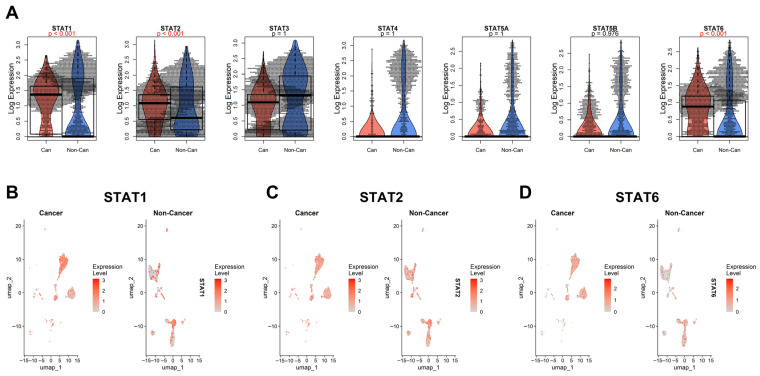
Single-cell RNA-seq analysis derived from the Puram dataset [45], comparing malignant epithelial cells (“Cancer”) and remaining cell populations (“Non-Cancer”). (**A**) Beeswarm/violin/boxplot representation of log-transformed expression levels for STAT1, STAT2, STAT3, STAT4, STAT5A, STAT5B, and STAT6. For each gene, cancer cells (red) are shown on the left and non-cancer cells (blue) on the right. Violin shapes depict distribution density; overlaid boxplots indicate median and interquartile range; dots represent individual cells. Adjusted *p*-values are displayed above each comparison (in red if significant). STAT1, STAT2, and STAT6 show significant global upregulation in cancer. (**B**) UMAP projection of single cells colored by STAT1 expression level in Cancer (left) and Non-Cancer (right) compartments. (**C**) UMAP projection colored by STAT2 expression. (**D**) UMAP projection colored by STAT6 expression. Color intensity reflects normalized expression levels. STAT1 and STAT2 show enrichment within malignant epithelial clusters, whereas STAT6 displays a more heterogeneous pattern with high expression in a minor non-cancer subpopulation, suggesting microenvironmental contribution.

To determine whether the STAT upregulation observed in bulk RNA-seq data reflects tumor-intrinsic transcriptional activation rather than stromal or immune infiltration, we analyzed single-cell RNA-seq data from head and neck squamous cell carcinoma [45]. Cells were classified into “Cancer” and “Non-Cancer” groups based on the original annotation (see Methods), allowing us to compare malignant epithelial cells against all remaining cell populations (Figure 6).

At single-cell resolution, STAT1 and STAT2 showed clear and consistent upregulation within malignant cell clusters compared with the aggregated non-cancer compartment. UMAP projections confirmed that their expression is enriched directly within tumor epithelial populations rather than being confined to infiltrating immune cells. These findings reinforce the notion that STAT1 and STAT2 activation is, at least in part, tumor-cell autonomous.

STAT6 displayed a more complex pattern. While global comparisons indicate significant upregulation in the cancer group, the beeswarm/violin/boxplot representation reveals that STAT6 expression is particularly elevated in a distinct minor subpopulation of non-cancer cells. This suggests that STAT6 activity may be enriched in specific stromal or immune subsets within the tumor microenvironment, potentially reflecting polarized immune states or cytokine-responsive niches.

It is worth mentioning that the “Non-Cancer” category in this dataset represents a heterogeneous mixture of cell types, including immune, stromal, endothelial, and possibly residual epithelial populations. It does not necessarily correspond to the precise normal cell of origin of the tumor, which remains undefined in this context. Therefore, while STAT6 appears significantly upregulated overall, its cellular specificity cannot be conclusively attributed solely to malignant epithelial cells in this dataset. Moreover, other foundational single-cell cancer datasets [48,49,50] did not reproduce this pattern; across those datasets, most STAT genes showed no consistent difference between malignant and non-malignant cells, with the main exception of STAT3 in nasopharyngeal carcinoma (Supplementary Figure S2), highlighting the need for better annotation and standardization for future single-cell datasets.

Future studies employing refined cell-type annotations and lineage-resolved comparisons (ideally contrasting tumor cells with their exact normal cellular counterpart) will be required to more precisely delineate STAT6’s tumor-intrinsic versus microenvironment-driven expression.

In the HNSCC [45] dataset, STAT1 and STAT2 were higher in malignant cells, whereas STAT6 showed a mixed pattern influenced by the non-malignant compartment.

### 3.5. Exploratory Analysis of Strand-Skewed STAT1 ChIP-Seq Read Distributions

**Figure 7:**
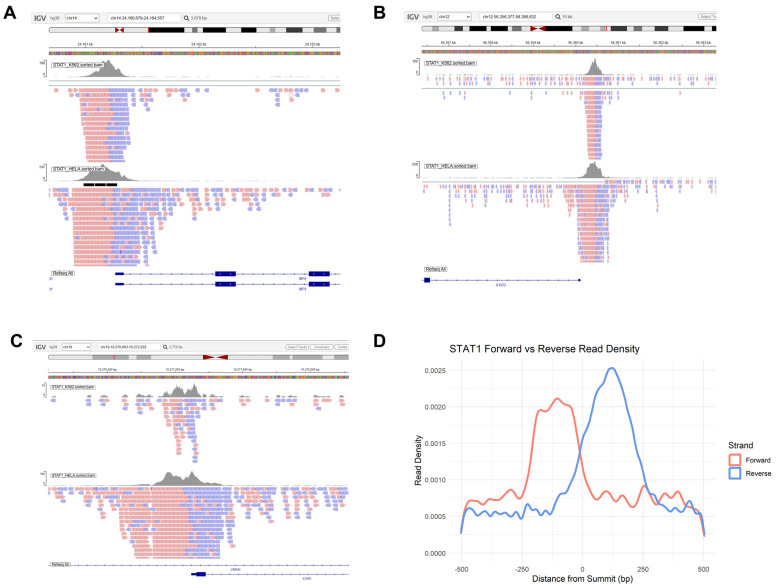
Genome-wide STAT1 binding profiles derived from interferon-stimulated HeLa cells [51] and K562 cells [52]. (**A**) IGV snapshot of a representative STAT1 binding locus at the IRF9 promoter. The upper tracks show read coverage density for K562 and HeLa cells. Individual reads are displayed below, with forward-strand reads in red and reverse-strand reads in blue, demonstrating strand-specific asymmetry relative to the peak summit. (**B**) IGV snapshot of a second representative locus at the STAT2 promoter, again demonstrating strand-biased distribution of reads around the binding peak. (**C**) IGV snapshot at the ICAM1 promoter locus, confirming identical non-canonical binding dynamics. (**D**) Aggregate metaprofile of STAT1 ChIP-seq signal centered on peak summits (±500 bp), combining both datasets. Forward (red) and reverse (blue) strand read densities are plotted separately. The curves display asymmetry, with offset enrichment between strands rather than symmetric accumulation around the summit. This pattern leaves open the potential for non-canonical, orientation-biased DNA recognition by STAT1.

Finally, we investigated STAT1 chromatin binding behavior by analyzing genome-wide ChIP-seq data derived from both interferon-stimulated HeLa cells [51] and K562 cells [52]. Visual inspection of canonical STAT1 target loci, including IRF9 and STAT2, which drive a positive feedback loop to amplify interferon signaling [61], and ICAM1, a key mediator of cell adhesion and immune infiltration [62], revealed a pronounced asymmetry between forward and reverse strand read distributions (Figure 7A–C). Rather than forming a perfectly centered pattern around the peak summit, reads showed strand-skewed distributions at these loci, indicating directional occupancy for STAT1. To evaluate this distribution globally, we calculated aggregate read density plots centered on all identified peak summits across both datasets. The resulting combined profile (Figure 7D) displays a clear spatial asymmetry. While this read distribution fundamentally reflects the standard bimodal strand shift inherent to single-end ChIP-seq fragment sequencing, it may also concurrently represent an asymmetric spatial occupancy of the transcription factor. STAT1 requires dimerization to effectively engage DNA [63]. Our data leaves open the possibility that the STAT1 dimer interacts with the Watson and Crick DNA fragments in an orientation-biased manner, further supporting prior structural evidence of half-site or non-palindromic binding [64,65]. Such asymmetric DNA recognition may expand the STAT1 regulatory repertoire, enabling interaction with composite regulatory elements or cooperative binding with additional transcription factors. This mechanistic flexibility may help explain why only a subset of STAT-bound genes undergo transcriptional modulation despite widespread chromatin occupancy [66,67,68]. Further studies should provide a clearer complement of epigenomic and transcriptomic data, asking whether oncogenic STAT activity may involve both quantitative upregulation and qualitative shifts in DNA-binding dynamics.

## 4. Discussion

In this study, we provide an integrated evolutionary, transcriptomic, network-level, single-cell, and chromatin-binding analysis of the STAT gene family across vertebrates and human solid tumors. Rather than focusing on individual STAT members, our results support a model in which the STAT family operates as an evolutionarily conserved but functionally plastic transcriptional module broadly activated in cancer.

Our phylogenetic reconstruction confirms the monophyletic expansion of vertebrate STATs, and the resulting topology structurally reinforces a functional dichotomy: STAT3 and STAT5 paralogs display high conservation reflecting strict endocrine pathway constraints, whereas immune-associated STATs show greater evolutionary divergence [24,26,57]. These evolutionary dynamics mirror the functional specialization observed in mammals. Specifically, the deep conservation of STAT3 and STAT5 highlights their fundamental roles in basic cellular proliferation and survival pathways that are universally hijacked across the 15 solid tumor types analyzed here (such as breast, prostate, and lung carcinomas) to drive autonomous oncogenic growth [12,16]. Conversely, the accelerated evolutionary divergence of the immune-associated STATs (STAT1, STAT2, STAT4, STAT6) generated a highly plastic signaling network originally shaped by host-pathogen arms races [69]. In cancers characterized by complex inflammatory microenvironments, such as head and neck squamous cell carcinoma (HNSCC) and lung adenocarcinoma, this evolutionary plasticity is likely co-opted by malignant cells. Rather than acting strictly as static tumor suppressors, the rapid adaptive potential of these immune STATs enables tumors to dynamically rewire their cytokine responses, adapt to chronic inflammation, and facilitate immune evasion [70,71].

At the transcriptomic level, our pan-cancer analysis reveals widespread upregulation of STAT genes across solid tumors. While STAT3 and STAT5 are established oncogenic drivers [13], our data extend this pattern to STAT1, STAT2, STAT4, and STAT6. The elevation of STAT1 and STAT2 is particularly notable, given their classical characterization as interferon-mediated tumor suppressors [70,72]. Increasing evidence indicates that chronic interferon signaling may promote tumor adaptation, therapy resistance, and immune modulation in advanced cancers [71]. This immune modulation is further exemplified by the role of STAT1 and STAT3 as key transcriptional regulators of CD274 (PD-L1), a primary driver of adaptive immune evasion and a critical target for current checkpoint inhibitor therapies [73]. The consistency of STAT upregulation across tumor types suggests that persistent cytokine signaling and inflammatory circuits constitute a systemic feature of malignant transcriptional reprogramming [14].

Network inference using *corto* [43] demonstrates that STAT regulons are broadly activated in tumors, indicating functional transcriptional engagement rather than passive overexpression. Because transcription factor activity depends on post-translational regulation and chromatin context [74,75], the coordinated enrichment of downstream targets confirms that STAT proteins act as active master regulators in cancer. In several tumor types, regulon activation exceeded the magnitude of individual gene upregulation, supporting a cooperative model in which STAT paralogs function as an interconnected module. Given their ability to form heterodimers and bind overlapping motifs [76,77], oncogenic STAT signaling likely emerges from combinatorial network behavior rather than single-gene dominance. The pharmacological relevance of this network is underscored by the clinical success of Janus kinase (JAK) inhibitors, as well as the advanced clinical development of direct small-molecule inhibitors and targeted degraders (PROTACs) specifically directed against STAT3 and STAT5 [78].

Recent comprehensive pan-cancer analyses have mapped the broad clinical and genomic landscape of the STAT family. For instance, a multi-omic profiling detailed the widespread genomic alterations, epigenetic modifications, and immunological correlates of the JAK-STAT pathway across numerous cancer types [79]. Similarly, Zhou et al. analyzed bulk transcriptomic data to establish the prognostic and immunological value of individual STAT genes, correlating their expression profiles with patient survival and tumor microenvironment infiltration [80]. While these foundational studies firmly established the clinical and biomarker relevance of STATs, they primarily relied on bulk expression correlations and broad genomic mapping. Our study builds upon these macroscopic findings by addressing how this widespread upregulation is functionally executed at the molecular level and separates cancer cells from paired physiological tissue. By applying master regulator analysis, we show that elevated STAT expression is accompanied by the activation of STAT downstream gene networks rather than existing as inert overexpression.

Single-cell RNA-seq analysis further refines this interpretation. STAT1 and STAT2 show clear enrichment within malignant epithelial populations, supporting a tumor-cell–intrinsic component of interferon pathway activation. STAT6, in contrast, displays a more complex pattern: although globally elevated in cancer, it is highly expressed in a minor subset of non-cancer cells, likely representing specific immune or stromal populations. This highlights both tumor-intrinsic and microenvironmental contributions to STAT signaling. The binary “Cancer vs. Non-Cancer” annotation available in current datasets aggregates heterogeneous cell types and does not necessarily represent the exact normal cell of origin. Future lineage-resolved single-cell analyses comparing tumor cells with their precise physiological counterparts will be necessary to fully disentangle tumor-driven from reactive STAT activation. More broadly, these results show the value of integrating pan-cancer cohorts with high-resolution single-cell data to achieve mechanistic clarity.

Finally, our ChIP-seq analysis reveals asymmetric STAT1 read distributions around peak summits, deviating from a perfectly centered model. This profile is likely driven by a combination of the standard technical bimodal shift of single-end sequencing and potential biological asymmetry. STAT proteins obligately dimerize to engage DNA [63], and structural and biochemical studies have shown that these dimers can bind half-sites or non-palindromic elements, particularly in alternative conformational states [4,65]. Thus, the observed asymmetry may partly stem from orientation-biased binding of the STAT dimer to the Watson and Crick strands. Asymmetric or context-dependent DNA recognition may help explain why only a fraction of STAT-bound loci undergo transcriptional modulation [28,81]. In cancer, such binding flexibility could expand the regulatory repertoire of STAT proteins beyond canonical interferon-stimulated genes, contributing to transcriptional plasticity within altered chromatin landscapes.

Our findings suggest that the STAT family represents an evolutionarily conserved transcriptional module that becomes broadly and functionally activated in cancer. Rather than acting solely as isolated oncogenes or tumor suppressors, STAT proteins operate as a coordinated regulatory network shaped by deep evolutionary constraints and tumor-specific signaling environments. Despite these findings, significant gaps in our understanding of STAT biology remain. While our ChIP-seq analysis suggests non-canonical, asymmetric DNA recognition dynamics, it is still unclear how this structural plasticity dictates definitive transcriptional outcomes. Future studies must investigate whether these asymmetric binding events represent half-site occupancy that facilitates cooperative binding with uncharacterized cofactors or composite regulatory elements. Furthermore, while our network analysis confirms widespread target engagement, the transition from pan-cancer transcriptomic profiling to patient-specific precision medicine remains a critical hurdle. Evaluating how these integrated STAT networks dynamically rewire in response to the emerging clinical therapies discussed above (such as targeted STAT degraders or immune checkpoint inhibitors) represents a vital next step for the field. Expanding integrative, multi-omics, and lineage-resolved studies will be essential to fully understand STAT-driven transcriptional architecture and to refine therapeutic strategies targeting JAK/STAT signaling in oncology.

## Data Availability

All data used in this study is publicly available, with sources stated in the text.

## Supplementary Materials

The following supporting information can be downloaded at https://www.mdpi.com/article/10.3390/genes17050547/s1. Supplementary Figure S1: Genomic Synteny of the STAT5 Locus in Monodelphis domestica. Visual representation from the NCBI Genome Data Viewer showing the tandem arrangement of the two STAT5A-like loci (canonical STAT5A and LOC100011470) on Chromosome 2 (NC_077228.1) of the Monodelphis domestica reference genome. Supplementary Figure S2: Single-cell transcriptomic profiling of STAT family expression (STAT1-STAT6) in malignant versus non-malignant cells across three independent cancer datasets. Violin plots display log-normalized expression values, with internal boxplots indicating median and interquartile ranges. (A) Melanoma microenvironment (GSE72056). (B) Breast cancer microenvironment (GSE75688). (C) Nasopharyngeal carcinoma microenvironment (GSE150430), highlighting the significant upregulation of STAT3 in cancer cells (*p* < 0.001).
