# Supplementary figures and images for: Integrated Evolutionary and Multi-Omic Analysis of STAT Family Activation Across Solid Tumors
Source: Genes (Basel). 2026 May 3;17(5):547. doi: 10.3390/genes17050547 (PMC13206669; doi:10.3390/genes17050547)

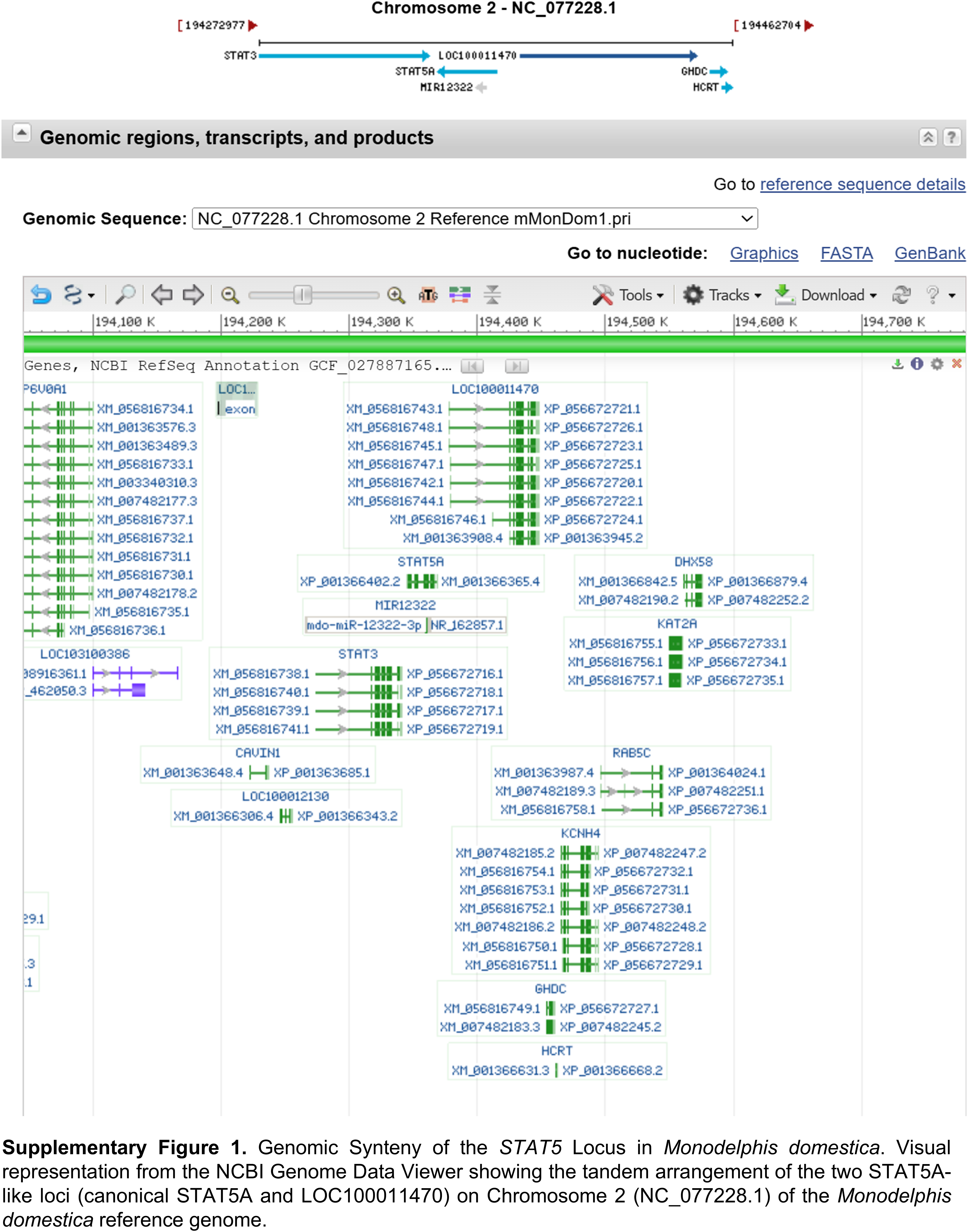

Supplement: Supplementary file 1 [file genes-17-00547-s001.zip › SuppFigure1.PNG]

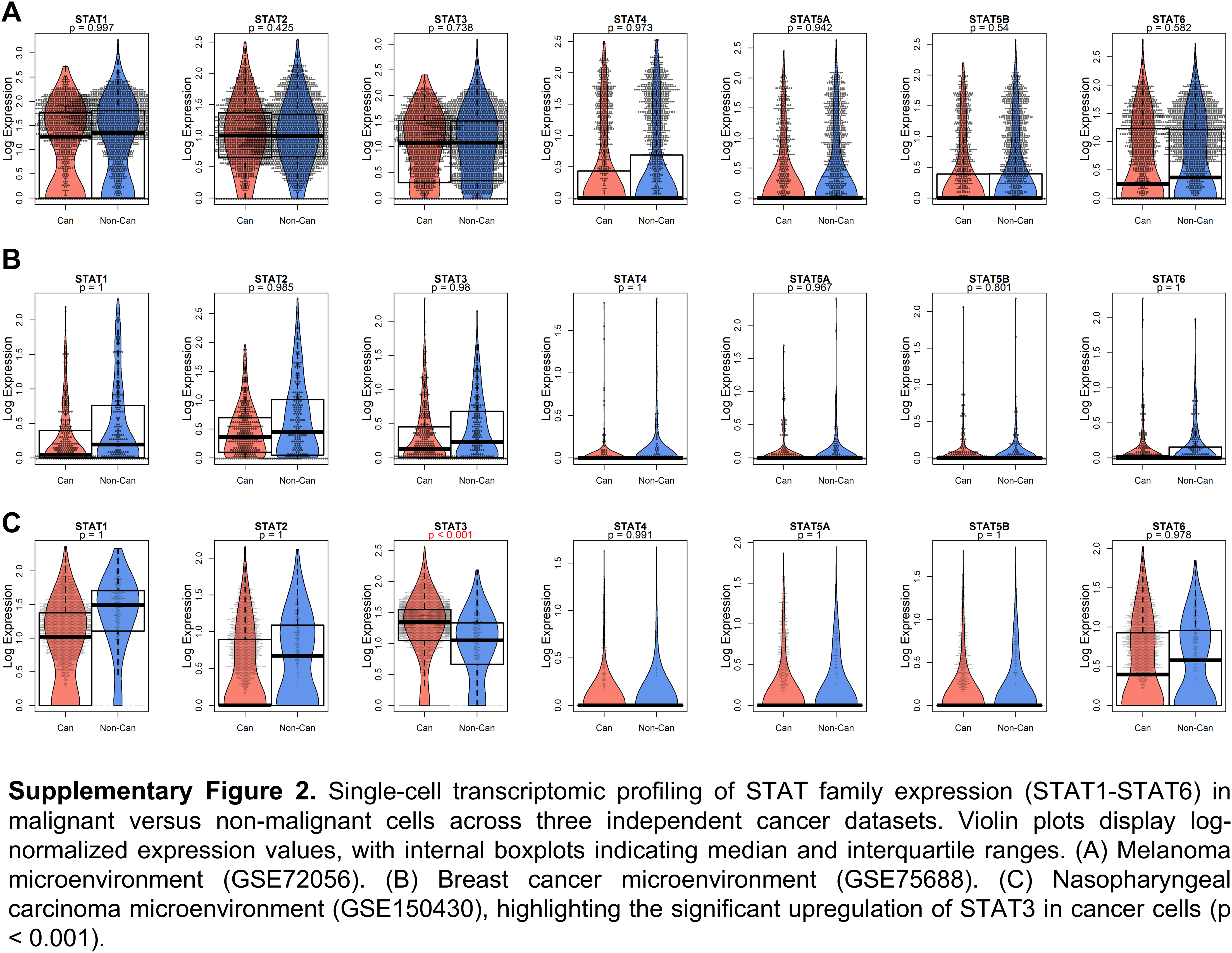

Supplement: Supplementary file 1 [file genes-17-00547-s001.zip › SuppFigure2.PNG]
